# Rab11 family expression in the human placenta: Localization at the maternal-fetal interface

**DOI:** 10.1371/journal.pone.0184864

**Published:** 2017-09-18

**Authors:** Elizabeth S. Taglauer, Patrycja A. Artemiuk, Sara R. Hanscom, Andrew J. Lindsay, Danielle Wuebbolt, Fionnuala M. Breathnach, Elizabeth C. Tully, Amir R. Khan, Mary W. McCaffrey

**Affiliations:** 1 Division of Newborn Medicine, Boston Children’s Hospital, Boston, Massachusetts, United States of America; 2 School of Biochemistry and Immunology, Trinity College, Dublin, Ireland; 3 Molecular Cell Biology Laboratory, School of Biochemistry and Cell Biology, Biosciences Institute, University College Cork, Cork, Ireland; 4 School of Medicine, Royal College of Surgeons Ireland, Dublin, Ireland; 5 Department of Obstetrics and Gynaecology, Royal College of Surgeons Ireland, Dublin, Ireland; Shanghai Jiao Tong University, CHINA

## Abstract

Rab proteins are a family of small GTPases involved in a variety of cellular processes. The Rab11 subfamily in particular directs key steps of intracellular functions involving vesicle trafficking of the endosomal recycling pathway. This Rab subfamily works through a series of effector proteins including the Rab11-FIPs (Rab11 Family-Interacting Proteins). While the Rab11 subfamily has been well characterized at the cellular level, its function within human organ systems is still being explored. In an effort to further study these proteins, we conducted a preliminary investigation of a subgroup of endosomal Rab proteins in a range of human cell lines by Western blotting. The results from this analysis indicated that Rab11a, Rab11c(Rab25) and Rab14 were expressed in a wide range of cell lines, including the human placental trophoblastic BeWo cell line. These findings encouraged us to further analyse the localization of these Rabs and their common effector protein, the Rab Coupling Protein (RCP), by immunofluorescence microscopy and to extend this work to normal human placental tissue. The placenta is a highly active exchange interface, facilitating transfer between mother and fetus during pregnancy. As Rab11 proteins are closely involved in transcytosis we hypothesized that the placenta would be an interesting human tissue model system for Rab investigation. By immunofluorescence microscopy, Rab11a, Rab11c(Rab25), Rab14 as well as their common FIP effector RCP showed prominent expression in the placental cell lines. We also identified the expression of these proteins in human placental lysates by Western blot analysis. Further, via fluorescent immunohistochemistry, we noted abundant localization of these proteins within key functional areas of primary human placental tissues, namely the outer syncytial layer of placental villous tissue and the endothelia of fetal blood vessels. Overall these findings highlight the expression of the Rab11 family within the human placenta, with novel localization at the maternal-fetal interface.

## Introduction

Rab proteins are a family of small molecular weight G-proteins that bind to a variety of downstream effectors in order to direct many key cellular functions. In their active state, Rabs have specific intracellular localizations and control specific biosynthetic and endocytic trafficking pathways, which define their function. Of particular interest to our research is the Rab11 subfamily, which is primarily localized to the pericentriolar endosomal recycling compartment and controls pivotal steps of endosomal vesicle trafficking. Rab11 has been studied in a variety of model systems with many important implications for human health and disease [1]. With their central role in vesicle trafficking, continued analysis of the Rab11 family within physiologic exchange interfaces can provide important insights into its function. The placenta is a major site of nutrient exchange in human physiology, maintaining maternal-fetal transfer throughout pregnancy. Interestingly, there have only been limited investigations of Rab proteins in placental cells. To begin addressing this, we examined the expression and localization of Rab11 subfamily members and their effectors in the human placenta.

The human placenta is established by a tightly controlled process of cell invasion into the maternal uterus during the first trimester of pregnancy. The main body of the placenta consists of villi that are lined by trophoblast cells, the main functional cell for maternal-fetal exchange. Placental villi are lined with a continuous outer layer of multinucleated syncytiotrophoblast cells as well as an inner cell layer of mononucleated cytotrophoblast cells. Nutrients are absorbed from the maternal blood surrounding the placenta, trafficked through these trophoblast cell layers and ultimately taken up by fetal blood vessels. These blood vessels form a network throughout the placenta that eventually leads to the umbilical cord and the developing fetus. The placenta is a valuable model system for cell biology, with several well-established cell lines along with accessible primary tissues for corollary investigation [2]. Human placental tissue is routinely discarded after delivery, and for research purposes, its collection is relatively straightforward with standard patient consent and physician coordination.

Prior studies have identified Rab proteins within placental cell lines, primarily as markers for intracellular vesicles of interest. Rabs 5 and 7 are found on a variety of vesicles within or derived from placental cells in studies focusing on HIV transmission, Salmonella infection and placental exosome formation [3–5]. In a study examining exocyst complex molecules, Rab11 was found within apical vesicle formations in placental cells [6]. However, a dedicated characterization of Rab proteins within human placental tissues has not been performed to date.

For our preliminary analysis, we chose to examine members of the endosomal Rab11 subfamily, namely Rab11a, Rab11b and Rab11c(Rab25) and the more distantly related endosomal Rab14 in a range of human cell lines by Western blotting. These proteins have a common binding partner RCP(also called Rab11-FIP1C), through which these proteins direct a number of critical cellular functions involving endosomal recycling [7]. More recent data has also implicated RCP in directing cell invasion [8, 9]. As these processes are prominent functional aspects of placental physiology, RCP and its common binding partners Rab11a, Rab25 and Rab14 were ideal candidates for an initial examination of Rab proteins within the human placenta. In this study, we identified a unique expression pattern of these proteins in placental cell lines as well as a prominent presence within key areas of primary human placental tissue samples. Overall, this work highlights the placenta as a novel interface for further for examination of Rab proteins in human tissue.

## Materials and methods

### Tissue collection and preparation

All tissues were collected in accordance with research ethics committee protocols approved by the Rotunda Hospital (REC-2015-012) and Trinity College Dublin (201600304). Following informed consent, normal term placental tissues were collected from n = 8 patients undergoing routine cesarean sections with an average gestational age of 38 weeks (+/- 0.26 SEM). Criteria for inclusion into the study for “normal term placentas” were pregnancies that progressed past 37 weeks gestation delivered by routine cesarian section (for breech presentation or repeat cesarian section) without evidence of intrauterine infection, preeclampsia/gestational hypertension or gestational diabetes and no fetal anomalies that could be prenatally detected via ultrasound/standard first trimester maternal serum screening. Tissues were fixed in 4% paraformaldehyde for 4 hours, soaked for 18–24 hours in 18% sucrose, embedded in Tissue-Tek O.T.C. cryopreservative medium (VWR) and frozen at -80°C. Tissue blocks were then sectioned via cryostat at 20 μm thickness for subsequent immunostaining.

### Cell culture

BeWo, Jar, H1299, and HeLa cells were obtained from the ECACC. BeWo cells were cultured in ATCC formulated Ham’s F-12K medium supplemented with 10% foetal bovine serum (Sigma), 100 U/ml penicillin/streptomycin, and 2 mM glutamine. JAR cells were cultured in RPMI-1640 medium supplemented with 10% foetal bovine serum (Sigma), 10mM Hepes, 1mM Sodium Pyruvate, 1500 mg/L Sodium bicarbonate, 4500mg/L glucose, 100 U/ml penicillin/streptomycin, and 2 mM glutamine. H1299 and HeLa cells were cultured in Dulbecco’s modified Eagle’s medium (DMEM) supplemented with 10% foetal bovine serum (Sigma), 100 U/ml penicillin/streptomycin, and 2 mM glutamine. All of the cell lines were maintained at 37°C in 5% CO_2_.

### Antibodies

Primary antibodies used for this study were Rab 11a (Invitrogen), Rab11b (Sigma) Rab 14 (Sigma), Rab 25 (Sigma), and RCP (Sigma). Additional details of primary antibodies including dilutions and Antibody Registration number are included in S1 Table. Secondary antibodies for immunofluorescence microscopy were Alexa 546 goat anti-chicken, Alexa 488 goat anti-mouse, and Alexa 647 goat anti-rabbit, all purchased from Life Technologies and used at a dilution of 1:400. For western blotting, IRDye800- and IRDye700- labelled goat anti-rabbit and goat anti-mouse secondary antibodies (Licor) were used at a dilution of 1:10,000.

### Immunostaining

For immunofluorescence microscopy of cultured cell lines, cells were seeded onto 10-mm glass coverslips followed by fixation with 4% paraformaldehyde and blocking/permeabilization with 0.05% saponin, 0.2% bovine serum albumin. Cells were then incubated with primary antibodies in blocking solution followed by extensive washing and application of secondary antibodies. Coverslips were mounted in Mowiol followed by confocal imaging. For immunofluorescence microscopy of placental tissue, cryosections of placental tissues were incubated for 1 hour at room temperature in PBS pH7.4 with blocking/permeabilization solution 0.2% Triton X-100 and 10% goat serum (Sigma). Goat serum was the host species of all secondary antibodies. Diluted primary antibodies were then added to slides for incubation at 4°C overnight. Following a series of washes secondary antibodies were added to slides for 1 hour at room temperature, followed by a second series of washes. For control staining of placental tissues, slides were incubated with only secondary antibodies (no primary antibodies). Antibodies were diluted in PBS pH 8 with 1% BSA, 0.3% Tween 20. All washes were performed with PBS pH 7.4. Following final washes, slides were coverslipped with Prolong Gold with DAPI and cured overnight prior to imaging.

### Western blotting

Tissue culture grown cells were washed twice in cold 1x PBS, then 100-300ul of cold RIPA buffer (10mM Tris-HCl pH7.6, 50mM NaCl, 50mM NaF, 1% NP-40, 1x Cocktail Stock (containing: Leupeptin, Chymostatin, Antipain and Aprotinin), 0.5mM AESBF, 1 Protease Inhibitor Cocktail Tablet (1 tablet per 10ml lysis buffer)) was added and cells were scrapped into 1.5ml tubes and lysed on ice for 15 minutes. The lysates were then passed 6 times though 26G needle and centrifuged at 14,000 rpm for 15 minutes at 4°C. Supernatant was transferred into fresh tube and quantified using a Bradford assay. 100ug of each sample was resolved by 12% SDS-PAGE, transferred onto nitrocellulose membrane, incubated with primary antibodies against Rab11a, Rab11b, Rab25, Rab14, and tubulin and subsequently incubated with secondary antibodies anti-mouse IRDye 680 and anti-rabbit IRDye 800.

For Western blotting of human placental tissues, 120mg of placental tissue from three different patients was homogenized and incubated with RIPA buffer (150mM NaCl, 1% Triton X-100, 0.5% Na deoxycholate, 0.1% SDS, 50mM Tris, pH 8.0) with protease inhibitors (Roche). Samples were centrifuged for 20min at 12,000 rpm, followed by collection of supernatant. Protein concentration was measured via standard Bradford assay. Equal amounts of sample were then separated by SDS-PAGE gel, transferred to a nitrocellulose membrane followed by incubation with antibodies against Rab11a, Rab14, Rab25 or β-Actin and secondary antibodies as described above. For both cell line and human placental tissue Western blots, membranes were scanned using the Odyssey Infrared Imaging System.

### Quantification of protein expression

Relative expression levels of Rab proteins in human cell lines were determined by densitometry using Image Studio software version 4.0. Densitometry values were then averaged and graphed along with standard deviation values calculated via the STDEV.S function in Excel, verison 2010.

### Imaging

For immunofluorescence analysis of the cell lines, images were acquired on a Zeiss LSM510 laser scanning confocal microscope, using 63X 1.4 NA Plan Apo objective. Post-acquisition analysis was performed with the Zeiss Zen 2009 software. Images of placental tissue immunofluorescence were acquired on a Leica SP8 scanning confocal with LAS X software, with Bitplane Imaris post imaging analysis software.

### Ethics approval

Rotunda Hospital Ethics Committee, REC-2015-012;Trinity SOM ethics Committee, # 201600304.

## Results

**Fig 1:**
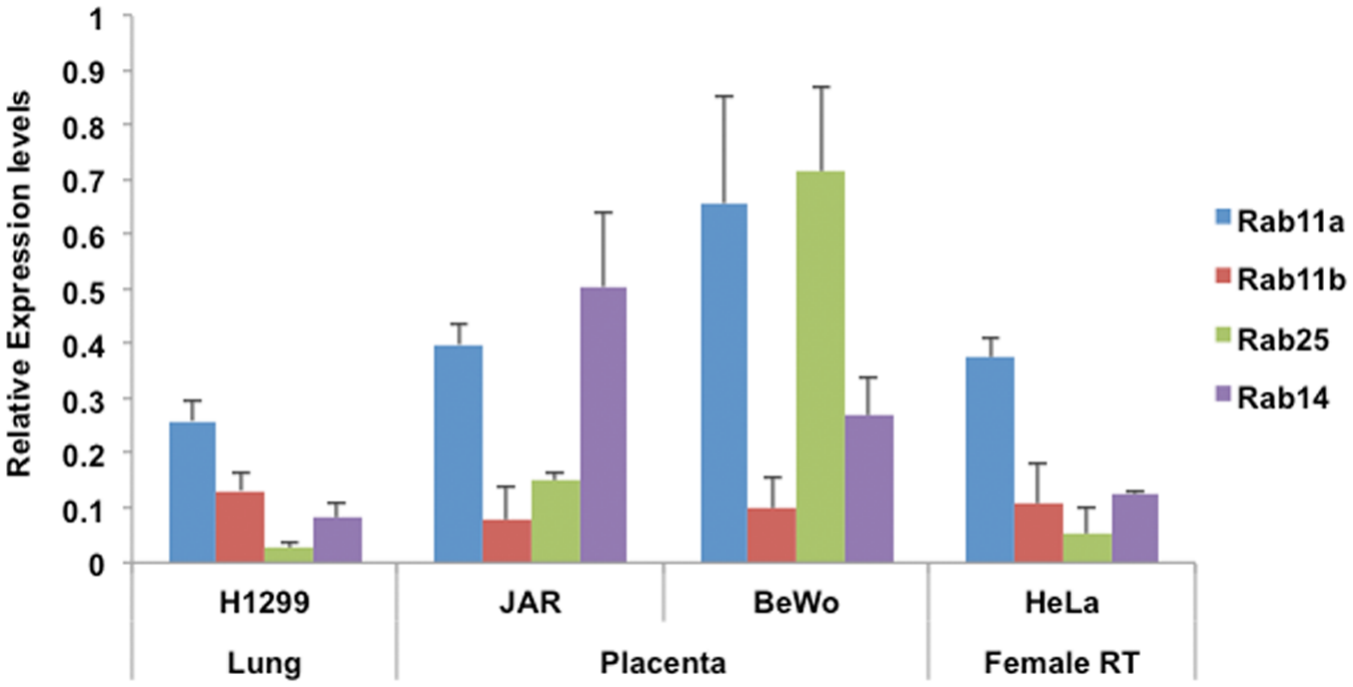
Survey of Rab11a, b, Rab 25 and Rab14 expression in human cell lines. The expression levels of Rab11a, Rab11b, Rab25 and Rab14 were analyzed by Western blot of lysates generated from several human cell lines. Combined data are shown as a histogram representing the expression levels of Rab11a, Rab11b, Rab 25(Rab11c) and Rab14 in various cell lines. Expression levels of each Rab were normalized to α-tubulin expression Colored bars represent mean of relative expression levels, error bars represent ± S.D; Rab11a/b, Rab25 n = 3, Rab14 n = 2).

We initially examined by Western blot analysis the expression levels of Rab11 family members in cultured placental cell types as compared HeLa cervical cancer and H1299 lung cancer cell lines (S1 Fig, Fig 1). Both HeLa and H1299 cells have been used as prior models for the study of Rab11 family proteins [10,11]. Interestingly, we found that Rab11a and Rab11c (henceforth referred to as Rab25) were abundantly expressed in BeWo cells compared with these other cell types, while Rab11b was detected at much lower levels in all of the cell lines tested (Fig 1). Given these results we decided to extend this analysis further. We next examined Rab14 in this range of cell types by Western blotting and found Rab14 to be well expressed in Jar and somewhat less in BeWo trophoblast cells, with its expression levels higher in these cell lines than in any of the other cell types tested (Fig 1).

**Fig 2:**
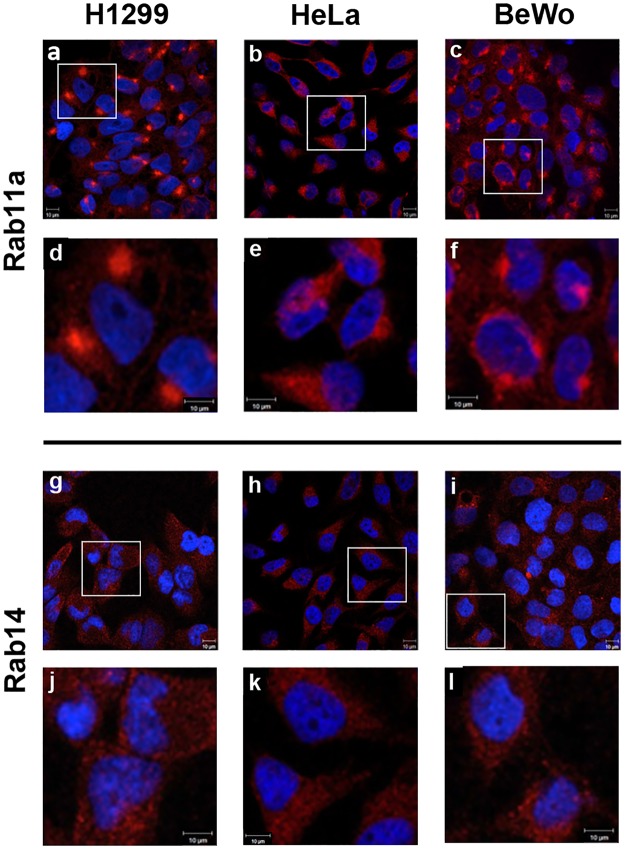
Comparative localization patterns of Rab11a and Rab14 in human cell lines. Rab11a and Rab14 localization was examined in cell lines H1299 (a,d,g,j), HeLa (b,e,h,k) and BeWo (c,f,i,l) by confocal immunofluorescence microscopy. Images a-c, g-i are representative fields of the indicated cells; images d-f, j-l are zoomed images. Ten micron scale bars included on each image.

**Fig 3:**
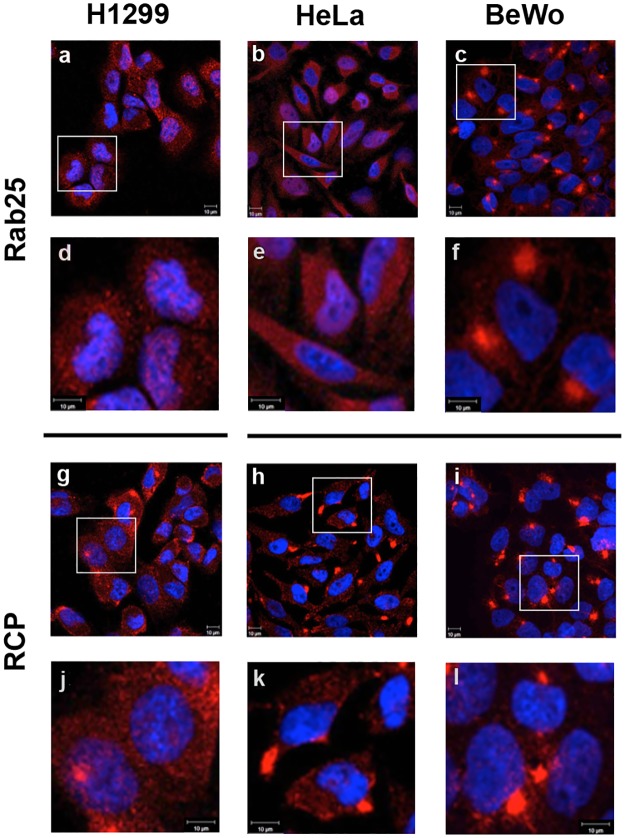
Comparative localization patterns of Rab25 and RCP in human cell lines. Rab25 and RCP localization was examined in H1299 (a,d,g,j), HeLa (b,e,h,k) and BeWo (c,f,i,l) cell lines by confocal immunofluorescence microscopy. Images a-c, g-i are representative fields of the indicated cells; images d-f, j-l are zoomed images. Ten micron scale bars included on each image.

Taking these combined data into account, we decided to investigate the localization patterns of these Rabs (Rab11a, Rab25 and Rab14), and their common effector RCP, by immunofluorescence microscopy of these *in vitro* cultured cell populations. For Rab11a, while H1299 cells expression was noted in both the cytoplasm and on endosomal structures, it was diffusely cytoplasmic in HeLa cells. In BeWo cells, Rab11a was both diffusely cytoplasmic as well as organized into notable peri-nuclear endosomal structures (Fig 2a–2f). Rab14 expression was noted to be cytoplasmic in all cell types examined (Fig 2g–2l). Further, in examining Rab25 (Fig 3a–3f), its distribution was also diffusely cytoplasmic in H1299/HeLa cells, but showed much more prominent peri-nuclear endosomal structures in BeWo cells. RCP was found both in the cytoplasm and condensed in peri-nuclear areas in BeWo cells, and also in H1299 and HeLa cultures (Fig 3g–3l).

**Fig 4:**
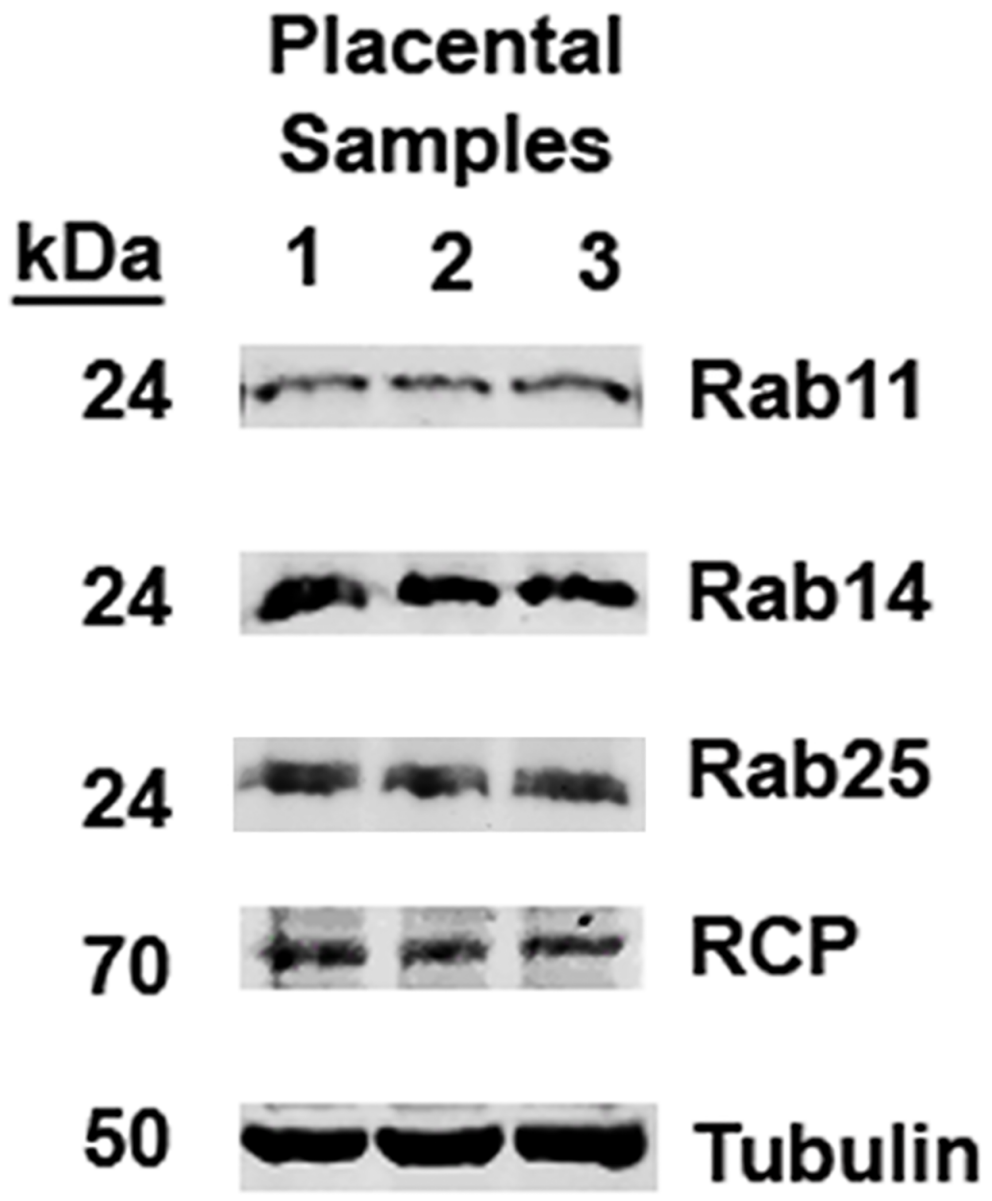
Immunoblot analysis of Rab11, Rab14 and RCP in human placental tissues. Tissue lysates were collected from the placentas of three different patients followed by Western blot analysis for Rab11, Rab14, Rab25 and their common binding partner RCP. kDa size for each target shown to the left of each blot image. Tubulin is included as a loading control.

We next examined the expression of these proteins in primary human placental tissue samples. Initial Western blot analysis of placental tissue lysates from three different patients showed expression of Rabs 11a, 14, 25 and RCP (Fig 4). We identified the expression of these proteins within all three term human placental samples. However, given the mixed cell populations present in whole tissue lysates, we chose to more specifically localize these proteins by means of immunofluorescence microscopy.

**Fig 5:**
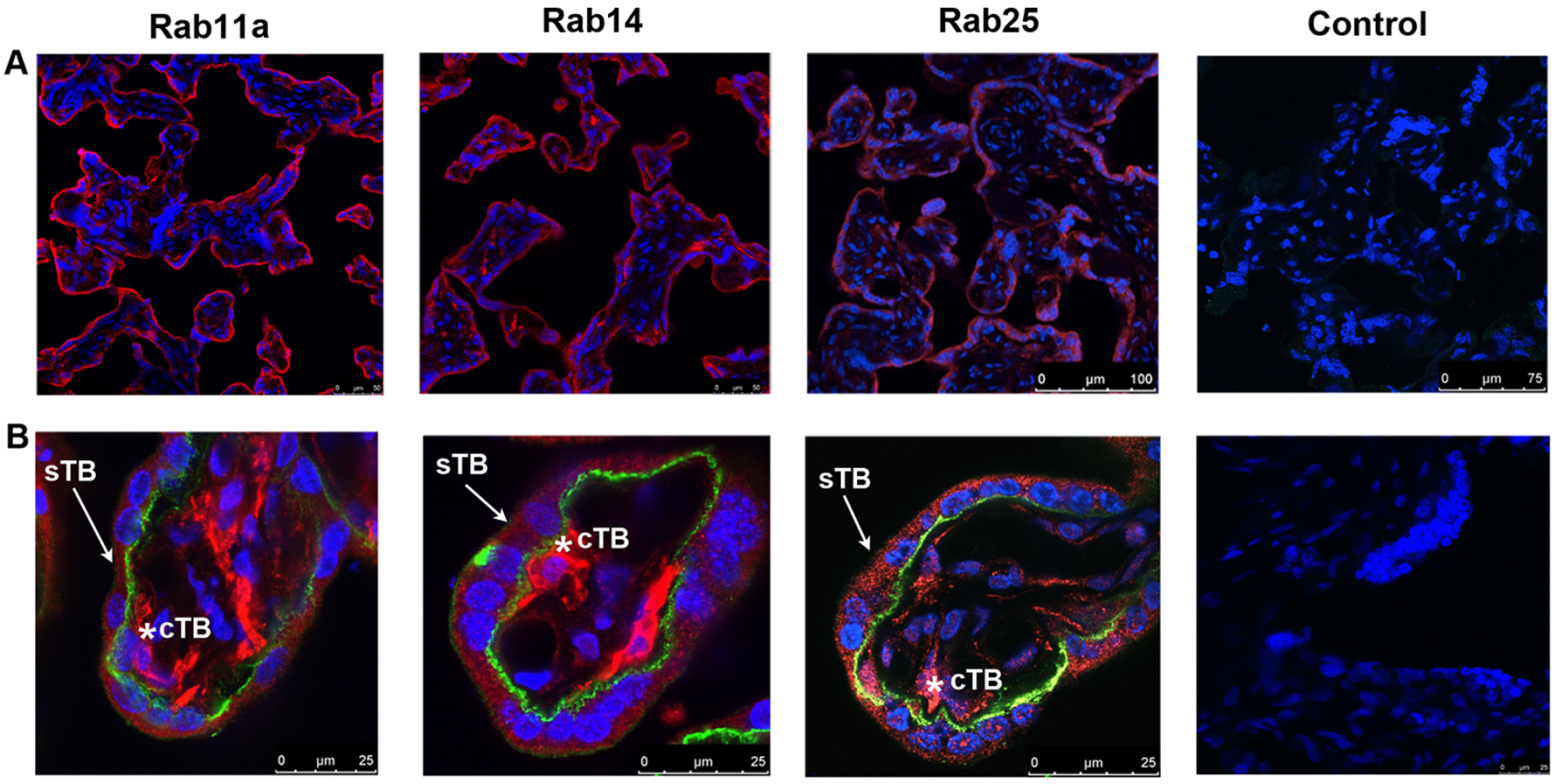
Localization of the Rab11 subfamily in human villous placenta. Placental tissue samples were collected from n = 8 patients. Expression of Rab11, Rab14 and Rab25 was analyzed in each placenta via immunofluorescence microscopy. Red label: Rab proteins, green label: E-cadherin, blue label: DAPI nuclear stain. Row A: representative low power views of the villous placenta Row B: representative high power views of individual placental villi. Asterisks indicate cytotrophoblast cells. Representative negative control staining images included for both high and low power views. Control samples are placental tissues incubated with secondary antibody only. For all images scale bars of 25–100 microns are included.

We thus conducted a survey of Rab proteins in primary placental tissues collected from eight different patients with healthy term pregnancies. Using immunofluorescence confocal microscopy we investigated the expression patterns of Rabs 11a, 14, 25. Major anatomical areas within the placental organ were examined, including decidual, villous and chorionic plate tissues. As first shown in Fig 5, expression of each Rab protein (shown in red) was strikingly present within the villous placenta, particularly in the outer syncytiotrophoblast (sTB) cell layer, demarcated by E-cadherin (green stain). They were also expressed within cell populations in the inner villous tissue (Fig 5 row B). Rabs 11a and 14 showed particularly prominent expression in the inner villous cell population, notably inside the E-cadherin border of the placental villous (Fig 5, row B). This expression was consistent amongst all samples evaluated.

**Fig 6:**
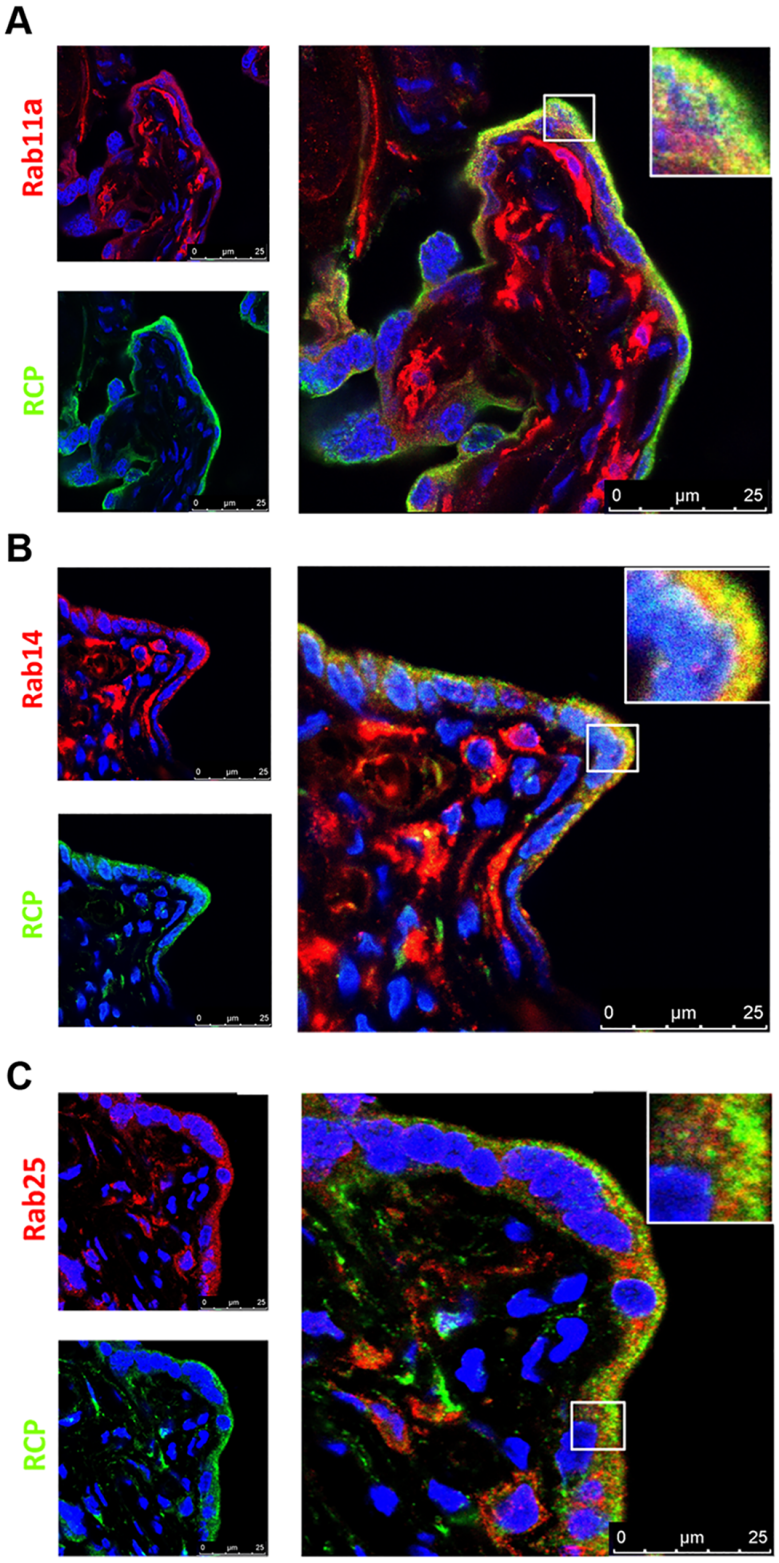
Co-localization of Rab11 subfamily with RCP in human placental villi. Double immunofluorescence microscopy of placental sample cohort for co-labeling of RCP with (A) Rab11, (B) Rab14, and (C) Rab25. Red label: Rabs, Green label: RCP, Blue label: DAPI nuclear stain). Representative individual staining shown on left with merged image on right. Inset outlined by white box shows magnified area of Rab/RCP co-localization as indicated by yellow color change. Twenty-five micron scale bars included on lower right of each image.

We next examined RCP expression and its co-localization with each of its binding partners. RCP was primarily expressed in sTB population with some notable expression in the inner cell populations as well (Fig 6). Of note, the main areas of co-localization with Rab proteins were in the outer sTB layer rather than inner villous cells.

**Fig 7:**
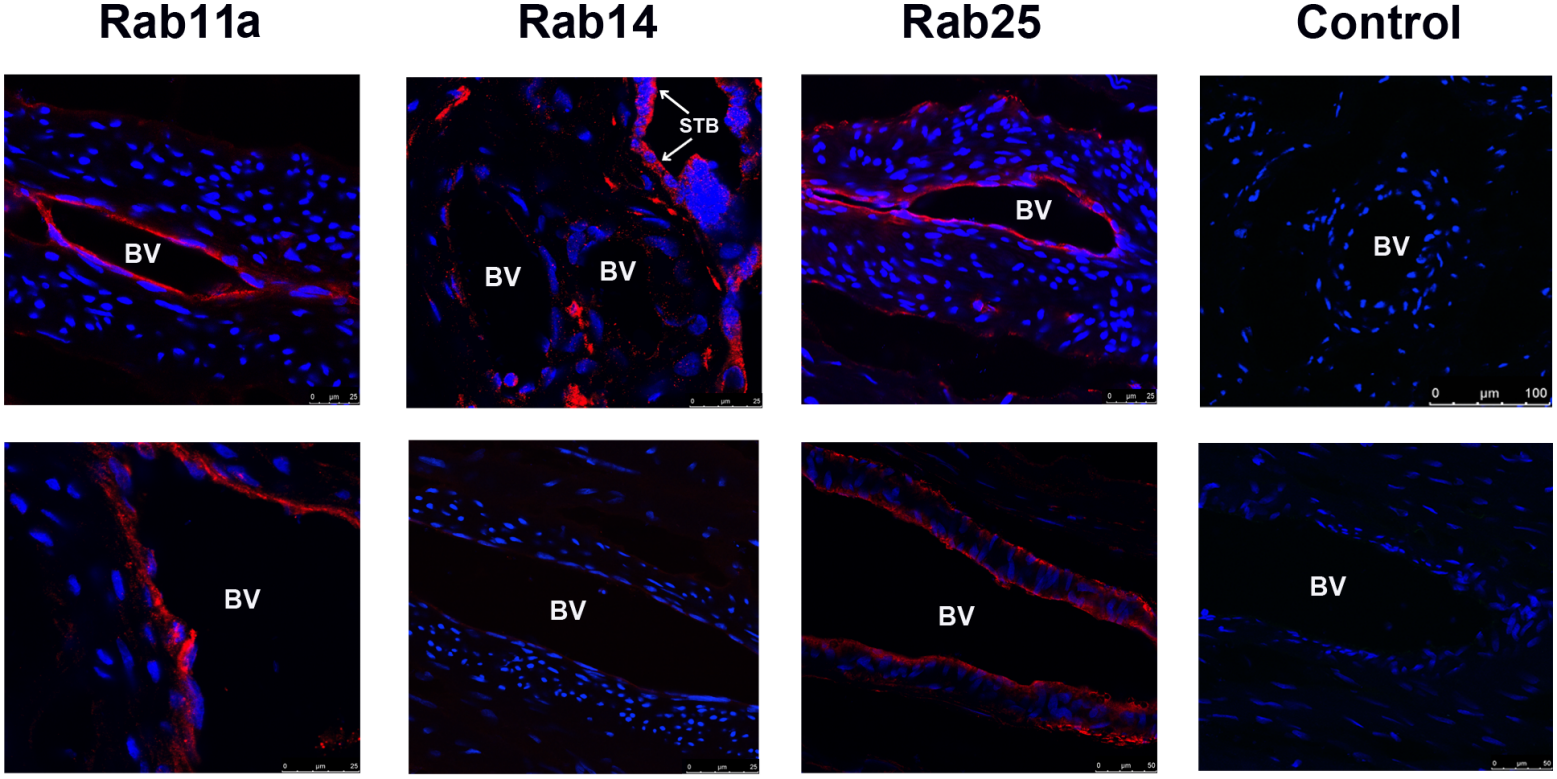
Expression of Rab11 subfamily in human placental blood vessels. Immunofluorescence microscopy analysis of Rab11, Rab14, and Rab25 expression in the fetal blood vessels within human placental samples. Red label: Rab proteins, blue label: DAPI nuclear stain. Two representative images of blood vessels for each Rab are displayed along with control images. Twenty-five micron scale bars included on lower right of each image. BV: Blood Vessel; STB: Syncytiotrophoblast.

**Fig 8:**
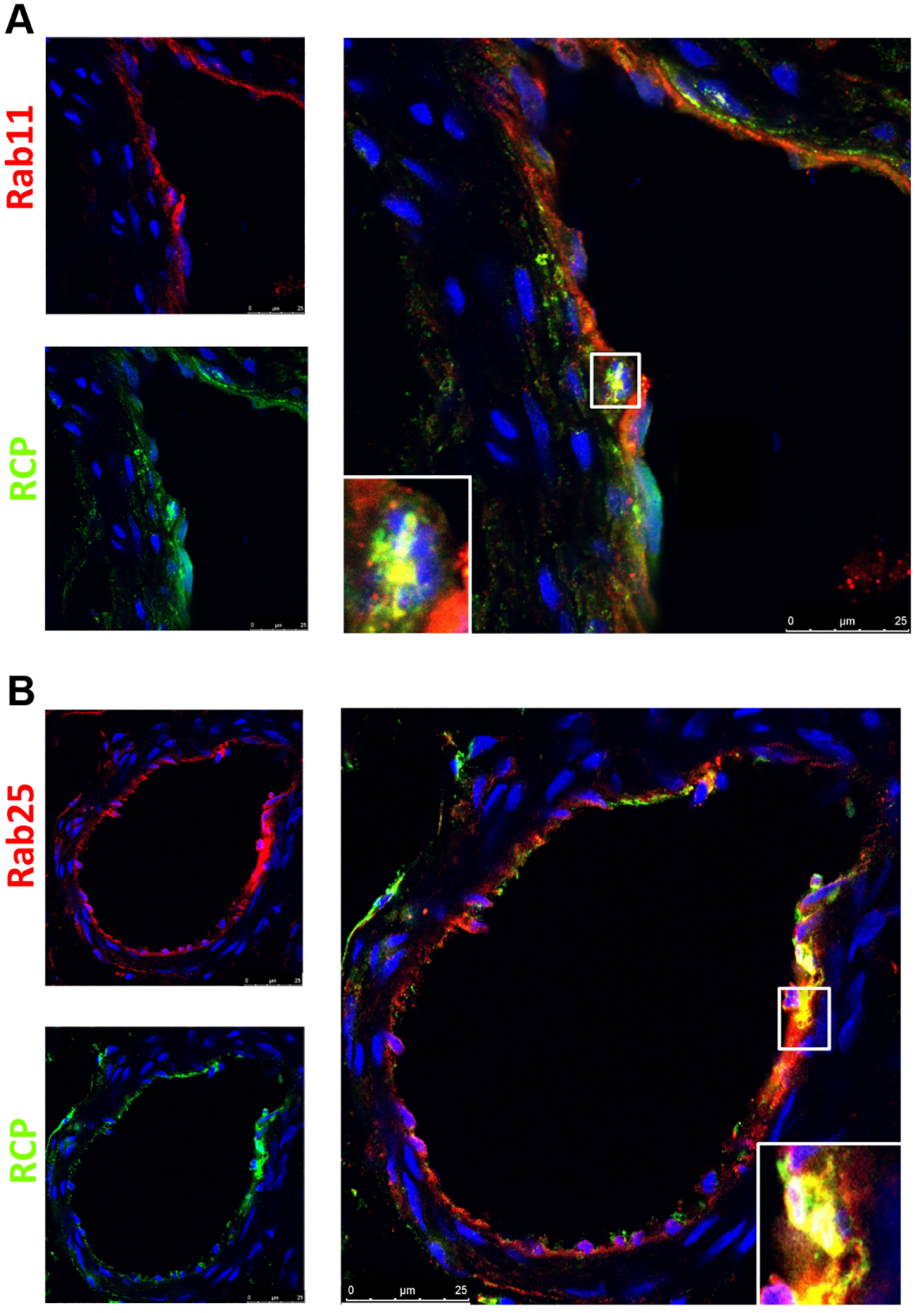
Co-localization of Rab11 and Rab25 with RCP in human placental blood vessels. Double immunofluorescence of placental sample cohort. Red label: Rab proteins, green label: RCP, blue label: DAPI nuclear stain. Individual staining shown on left with merged image on right. Inset outlined by white box shows magnified area of Rab/RCP co-localization as indicated by yellow color change. Micron scale bars included on lower right of each image.

In a further survey of placental tissues, we also identified prominent Rab expression in the endothelium of fetal blood vessels. Rab 11a and 25 endothelial expression was consistent throughout all tissues examined (Fig 7). Interestingly, Rab14 was not detected in fetal endothelium. This is demonstrated in the low power image of prominent Rab14 expression in sTB with comparatively minimal expression in endothelium within the same tissue (Fig 7). With Rab11a/25, we also identified concomitant localization of RCP in fetal blood vessel endothelium (Fig 8).

## Discussion

In this preliminary investigation of Rab proteins in placental cells and tissues, we have demonstrated expression of Rabs 11a, 14 and 25 plus their common effector RCP at the human maternal-fetal interface. These proteins are prominently expressed in cultured placental cells with unique distribution in key areas of primary human placental tissues. While Rab proteins and their effectors have been studied extensively *in vitro*, continued analysis of their expression within human tissues is an informative baseline for the ongoing study of these trafficking molecules. By establishing the expression of Rab11family proteins within the BeWo cell line (Figs 1–3), this study helps lay the foundation for further analysis of Rab proteins utilizing placental cells.

**Fig 9:**
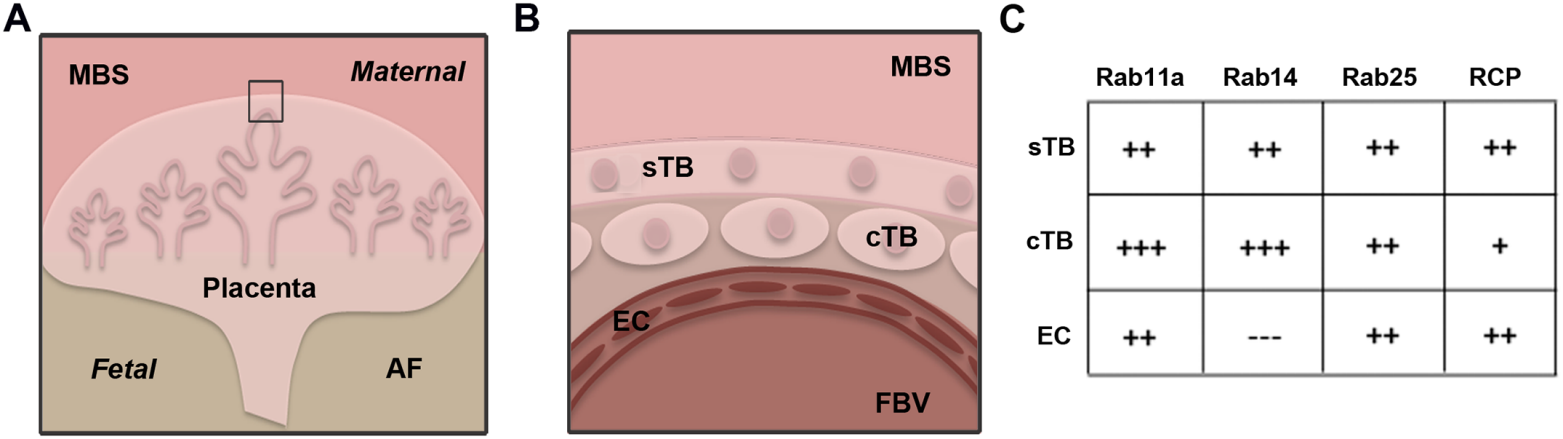
Summary of Rab11 family protein expression in human placenta. A. Diagram of human fetal placenta at the interface of the maternal uterine blood space (MBS) and the fetal amniotic fluid cavity (AF). B. Enlarged view of placental cell layers within villous tissue highlighting the outer syncytiotrophoblast layer (sTB), the inner cytotrophoblast layer (cTB) juxtaposed with fetal blood vessels (FBV) lined by endothelial cells (EC). C. Table summarizing localizatoin of Rabs 11a, 14, 25 and RCP within sTB, cTB and EC; (+) or (-) indicating the observed degree of expression within these cell types.

BeWo cells are a widely used cell line for the analysis of trophoblast function, of which Rab proteins may have a key role [12, 13]. For example, BeWo cells have been used to study iron transport [14], which is a Rab-mediated intracellular trafficking pathway [15]. Another advantage of using placental cell lines to study Rab proteins is having accessible material for corollary *in situ* studies in primary human tissue. Utilizing both Western blot and immunofluorescence, we demonstrated the expression of Rabs 11a, 14 and 25 and RCP in primary human placental tissue samples among a cohort of eight patients with healthy term pregnancies. Notably, these proteins were expressed in key functional areas of the human placenta, summarized in Fig 9.

We identified Rab 11a in syncytiotrophoblast cells in accordance with prior findings [6]. We also identified novel localization of Rab14 and Rab25 within the syncytiotrophoblast cell layer (Fig 5). The expression of these three proteins was prominent within this layer among all samples examined, lining the surface of the fetal placenta. The syncytiotrophoblast is the outermost cellular interface of the fetal placenta and is a highly active endocytic interface for the import of materials from the maternal blood. With their common role in various key aspects of endocytosis [7], the prominent and consistent expression of these Rab GTPases in this location suggests that placental tissue could be an useful tissue for future analysis of these proteins.

We further identified expression of all three Rab proteins in the inner cytotrophoblast cell population, with Rab14 and 25 showing particularly strong expression in these cells (Fig 5). Cytotrophoblast cells serve as a reservoir for the syncytiotrophoblast layer, which is continuously shed throughout pregnancy. From a methodology standpoint, cytotrophoblast cells are one of the few primary placental cell populations that have been successfully isolated for *in vitro* manipulation [16, 17]. The expression of these Rab proteins in this population was an important finding for the possibility of further study utilizing a placental model system. Syncytiotrophoblast cells, due to their structure, are much more technically challenging to isolate from placental tissues. However, primary cytotrophoblast cells can be induced to syncytialize and take on sTB characteristics *in vitro*, allowing study of this cell population in various stages of development [18].

Localization studies of RCP within the villous placenta demonstrated a varied distribution (Fig 6). RCP was strongly expressed within the outer STB layer where it co-localized with Rabs 11a and 14. RCP co-localization was less consistent with Rab25 (Fig 6). Interestingly, RCP was less prominent among the inner cytotrophoblast populations. These results suggest that RCP might be induced as cytotrophoblast cells differentiate to syncytiotrophoblasts. With the methods currently available, the placenta could be an interesting primary tissue model system in which the components that govern RCP expression/induction could be studied in cells as they differentiate.

Co-localization of Rabs 11a, 14 and 25 with their effector RCP within the syncytiotrophoblast cell layer is an important first step toward further investigation of these proteins at the maternal-fetal interface. Within the villous placenta (Fig 9A), the sTB layer is the active interface with the maternal blood space (Fig 9B). Syncytiotrophoblast cells carry out several functions including hormone production, nutrient transport and maternal immunoglobulin uptake [2]. As key regulators of vesicular dynamics, Rab proteins are known to aid in these same functions in other cell types [1,7,19]. It could be possible that within the placenta, Rab11 proteins and their common effector, RCP work within the syncytiotrophblast layer to help carry out these varied placental functions. However, as the antibodies utilized in this study do not differentiate between active and inactive states of these Rab GTPases, further analysis is required to more clearly characterize their state of activity within this tissue interface.

Finally, we identified Rabs 11a and 25 in the endothelium of fetal blood vessels throughout placental tissues (Fig 7), which co-localized with endothelial RCP expression in these vessels (Fig 8). Interestingly, Rab14 was not found in fetal blood vessel endothelia, its expression was restricted to the trophoblast cell populations of the villous placenta. The functional significance of this finding is, at present, unclear.

Prior studies have examined Rab11 function in human endothelial cells both from the umbilical cord and lung. In these models, Rab11 appears to mediate secretion of soluble vascular endothelial growth factor receptor 1(sFlt-1) [20] and help with junctional re-annealing after vascular inflammation [21]. Our work compliments these *in vitro* studies by localizing Rab11 as well as Rab25 *in situ* within the fetal vascular network of the human placenta. While Rab25 has been primarily characterized in epithelial cells, placental tissue could be a novel interface where this protein could be studied within an endothelial cell population. Several protocols have been developed for the isolation of primary human vein endothelial cells. Interestingly, two recent studies utilized primary human vein endothelial cells to study the function of Rab proteins; Rab11 as mentioned above [20] and Rab3B, which may participate in IgG antibody uptake [19]. This work, in conjunction with our *in situ* findings, presents an additional placenta-based model system that can be utilized for the further study of Rab trafficking molecules.

Given these preliminary findings, we propose that additional study of Rab proteins and their downstream effectors at the maternal-fetal interface is warranted. With Rab expression in multiple areas, the human placenta is an informative tissue to study these proteins in diverse cell types in the same organ/physiologic environment. Several models utilizing primary placental tissues are already established for functional studies including isolation and manipulation of primary cytotrophoblast cells and human umbilical vein endothelial cells as discussed above. Additionally, placental villous explants are a tool to study active trophoblast function in culture, particularly with studies of placenta-derived microparticles [22], on which some Rab proteins have been previously identified [5]. Furthermore, cell invasion assays utilizing primary trophoblast cells are also a well-developed model system to study. These may be particularly useful for the study of Rab25, which directs integrin recycling vesicle localization to promote cell migration and RCP, which mediates endosomal recycling of N-cadherin to influence cell motility [8, 9].

Overall, these results identify Rab protein expression within a physiologically important human tissue interface. Further, the study of Rab proteins within the placenta may also have broader applications. The placenta is an organ involved in dynamic processes and it has multiple roles throughout pregnancy. Each of these diverse processes is accomplished by underlying intracellular functions, in which Rab proteins may have an active role. An essential advantage of this system is the ability to work with a primary tissue with many functions that can be potentially applied to a broad range of human physiology.

## Supporting information

S1 Table Primary antibody information. Primary antibodies used in western blot analysis and immunofluorescence microscopy of cultured cells or placental tissue. Dilutions for various experiments indicated by the following superscripts: WB: Western blot, C: immunofluorescence microscopy of cultured cells; T: immunofluorescence microscopy of human placental tissues.
(TIF)

S1 Fig Western blot analysis of Rab11a, b, Rab 25 and Rab14 expression in human cell lines. A variety of human cell lines were lysed and the expression levels of Rab proteins; (A) Rab11a, (B) Rab11b, (C) Rab25 (Rab11c) and (D) Rab14 were analysed by Western blotting. Representative blots are shown for each Rab protein along with the α-tubulin loading controls.
(TIF)
