# Supplementary figures and images for: Rab11 family expression in the human placenta: Localization at the maternal-fetal interface
Source: PLoS One. 2017 Sep 18;12(9):e0184864. doi: 10.1371/journal.pone.0184864 (PMC5602629; doi:10.1371/journal.pone.0184864)

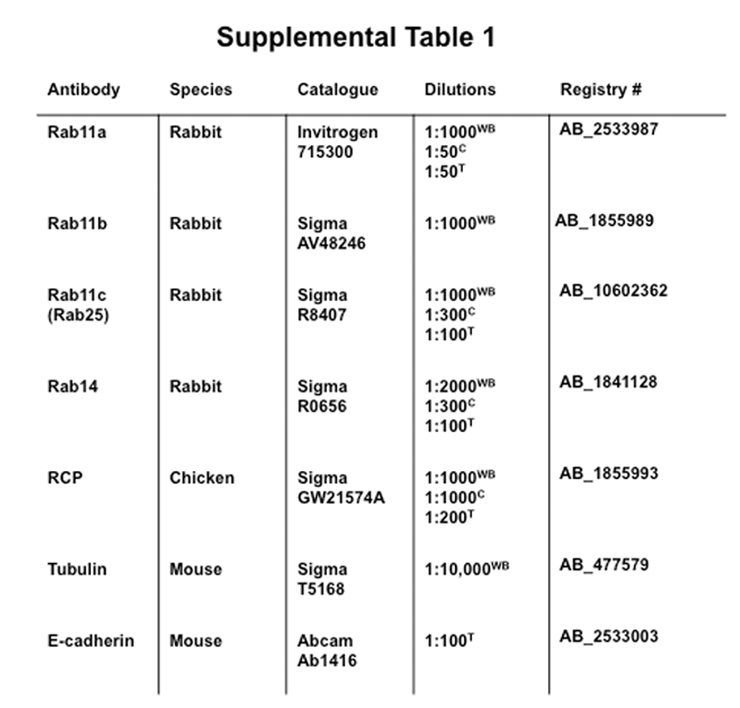

Supplement: S1 Table — Primary antibodies used in western blot analysis and immunofluorescence microscopy of cultured cells or placental tissue. Dilutions for various experiments indicated by the following superscripts: WB: Western blot, C: immunofluorescence microscopy of cultured cells; T: immunofluorescence microscopy of human placental tissues. (TIF) [file pone.0184864.s001.tif]

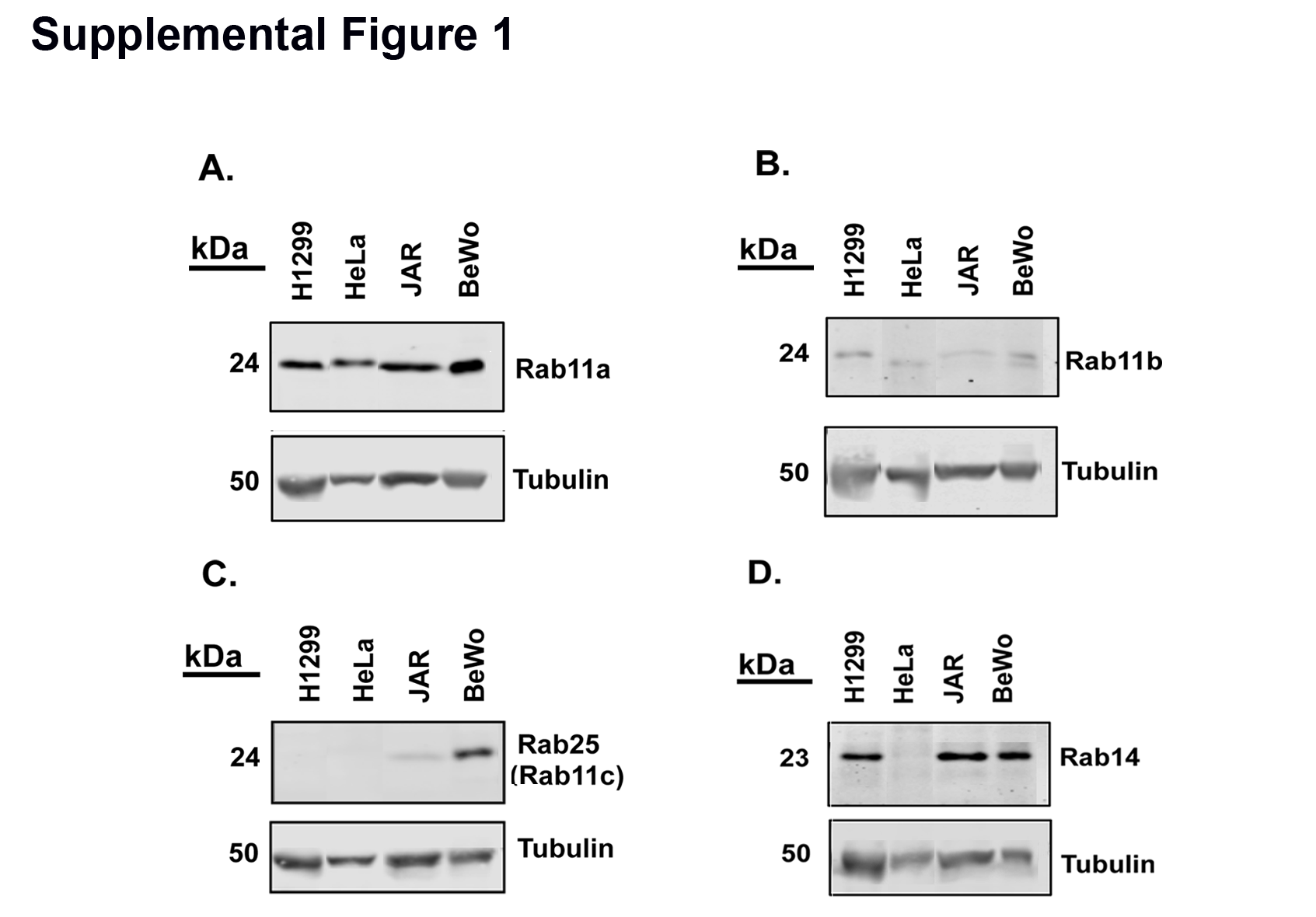

Supplement: S1 Fig — A variety of human cell lines were lysed and the expression levels of Rab proteins; (A) Rab11a, (B) Rab11b, (C) Rab25 (Rab11c) and (D) Rab14 were analysed by Western blotting. Representative blots are shown for each Rab protein along with the α-tubulin loading controls. (TIF) [file pone.0184864.s002.tif]
